# 

**Published:** 1869-09

**Authors:** 


### INDEX OF HALL'S HEALTH TRACTS.

Containing 320 Health Tracts on the following subjects, with a steel engraving of the Editor, sent post-paid for $2.50.

Aphorisms Physiolog'l.
Apples.
Antidote to Poisons,
Acre, One.
Apoplexy.
Burying Alive.
Baths and Bathing.
Beards.
Bites and Burns.
Backbone.
Beauty a Medicine.
Best Day.
Baldness.
Burning to Death.
Bilious Diarrhea.
Balm of Gilead.
Bread.
Cold Cured.
" Neglected.
" Avoided.
" Nothing but a.
" In the Head.
" How Taken.
" Catching.
Consumption.
Coffee-Drinking.
Catarrh.
Checking Perspiration.
Centenarian.
Child-Bearing.
Children's Eating.
" Feet.
Children Corrected.
" Dirty.
Coal-Fires.
Cute Things.
Coffee Poisons.
Clothing, Flannel.
" Woolen.
" Changing.
Cholera.
Clergymen.
Cancer.
Corn-Bread.
Convenient Knowledge.
Charms.
Cooking Meats.
Cheap Bread.
Church Ventilation.
Cough.
Dyspepsia.
Drinking.
Diet for the Sick.
Deafness.
Debt, a Death!

Diarrhea.
Death.
Dying Easily.
Drowning.
Diptheria.
Dysentery.
Disinfectants.
Death Rate.
Deranged.
Digestibility of Food.
Dirty Children.
Drugs and Druggery.
Eyes, Care of.
" Weak.
" Failing.
Erect Position.
Eating.
" Wisely.
Eat, How to.
" What and when to.
Eating Habits.
" Great.
" Curiosities of.
" Economical.
Emanations.
Elements of Food.
Fruits, Uses of.
Flannel Wearing.
Follies, Fifteen.
Fire-Places.
Fifth Avenue Sights.
Food and Health.
Feet.
Fetid Feet.
Food, Nutritiousness of.
" its Elements.
Greed of Gold.
Genius, Vices of.
Great Eaters.
Gruels and Soups.
Hair Wash.
Health a Duty.
" Observances.
" Essentials.
" Theories.
Hydrophobia.
Headache.
Housekeeping.
Housewifery.
Household Knowledge.
Habit.
Home, Leaving.
Happiest, Who are.
Hunger.
Inconsiderations.

Ice, Uses of.
Inverted Toe-Nail.
Insanity.
In the Mind.
Kindness Rewarded.
Law of Love.
Longevity.
Life Wasted.
Loose Bowels.
Leaving Home.
Logic Run Mad.
Medicine Taking.
Memories.
Music Healthful.
Milk, its Uses.
Miasm.
Marriage.
Morning Prayer.
Month Malign.
Mental Ailments.
Mind Lost.
Medical Science.
Neuralgia.
Nursing Hints.
Nervous Debilities.
Old Age Beautiful.
One Acre.
One by One.
Obscure Diseases.
Precautions.
Presence of Mind.
Premonitions.
Private Things.
Poisons and Antidotes
Pain.
Peaceless.
Preserves.
Parental Trainings.
Philosophy.
Physiological Items.
Posture in Worship.
Physician, Faithless.
Popular Fallacies.
Punctuality.
Providence.
Rheumatism.
Read and Heed.
Resignation.
Restless Nights.
Recreation, Summer
Restlessness
Sitting Erectly.
Shoes Fitting.
Sabbath.
Sour Stomach.

Sick Headache.
Sunshine
Skating.
Suppers, Hearty.
Soldiers Remembered.
" Cared for.
" Health.
" Items.
" All.
Serenity.
Sores.
Sunday Dinners.
Small-Pox.
Sleep and Death.
Spot the One.
Specifics.
Spring-Time.
Summer Drinks.
Sickness not Causeless.
Sayre, the Banker.
September Malign.
Summer Mortality.
Stammering.
Soups and Gruels.
Sick School-Girl.
Stomach's Appeal.
Sleep.
Summerings.
Study, Where to.
Salt Rheum.
Sordid.
Traveling Hints.
Three Ps.
Teeth.
Toe-Nail, Inverted.
Thankful Ever.
Urination.
Valuable Knowledge.
Vaccination.
Ventilation.
Vermin Riddance.
Winter Rules.
Walking.
Warning Youth.
Woman's Beauty.
Whitlow.
Whitewashes.
Worth Remembering.
Worship, Public.
" Posture in.
" Without Price
Weather Signs.
Weather and Wealth.
Warmth and Strength.
Worth Knowing.

Address "Hall's Journal of Health," No. 2 West 43d St., New-York. ($1.50 a Year.)

